# Significant Response to Denosumab Yet with Severe Rebound Hypercalcemia in a 9-Year-Old Boy with Aneurysmal Bone Cyst: A Case Report

**DOI:** 10.3390/children12111524

**Published:** 2025-11-11

**Authors:** Laurence Allain, Sarah Elbaz, Sayanthen Sathyakumar, William Le Gallou, Christina Coleman, Hallie Coltin, Dardye Eugène, Abha Gupta, Sebastiano A. G. Lava, Samuele Renzi

**Affiliations:** 1 Faculty of Medicine, Laval University, Quebec City, QC G1V 0A6, Canada; 2 Division of Pediatric Hematology Oncology, CHUL-Laval, Quebec City, QC G1V 0A6, Canada; 3 Montreal Children’s Hospital, Montréal, QC H4A 3H9, Canada; 4 Department of Pediatric Hematology/Oncology, CHU Sainte Justine, Université de Montréal, Montréal, QC H3C 3A7, Canada; 5 Division of Pediatric Endocrinology, CHUL-Laval, Quebec City, QC G1V 0A6, Canada; 6 Department of Pediatrics, Laval University, Quebec City, QC G1V 0A6, Canada; 7 Princess Margaret Cancer Centre, University Health Network, Toronto, ON M5G 1X6, Canada; 8 Adolescent & Young Adult (AYA) Oncology Program, Princess Margaret Hospital, Toronto, ON M5G 1X6, Canada; 9 Paediatric Cardiology Unit, Department of Pediatrics, Centre Hospitalier Universitaire Vaudois and University of Lausanne, Rue du Bugnon 50, 1011 Lausanne, Switzerland; 10 Division of Clinical Pharmacology and Toxicology, Institute of Pharmacological Sciences of Southern Switzerland, Ente Ospedaliero Cantonale, 6900 Lugano, Switzerland

**Keywords:** aneurysmal bone cyst, tumor, rare pediatric tumors, bones, denosumab, targeted therapy, hypercalcemia, acute kidney injury, toxicity

## Abstract

A 9-year-old boy presented with edema of the left cheek. He was diagnosed with a large aneurysmal bone cyst of the mandibular bone. Off-label treatment with denosumab for 17 months resulted in a significant reduction in the lesion. Five months after discontinuing denosumab, the patient developed severe rebound hypercalcemia and acute kidney injury, which resolved with corticosteroids. Two years after treatment discontinuation, the lesion continues to decrease in size. This case highlights the efficacy of denosumab as an off-label treatment for aneurysmal bone cysts, though it can be associated with hypercalcemia and acute kidney injury. We conclude that the use of denosumab should be considered on a case-by-case basis, with regular monitoring for side effects.

## 1. Introduction

Aneurysmal bone cysts (ABCs) are benign bone lesions typically affecting children and young adults [1], with nearly 80% of these lesions occurring within the first two decades of life [2]. Although these lesions can occur in any bone, they more commonly arise in the metaphyseal region of long bones, around the knee and vertebral column [3]. The presence of these lesions in the jaw bones is exceedingly rare, accounting for less than 2% of aneurysmal bone cysts [4,5]. At the microscopic level, aneurysmal bone cysts are characterized by their blood-filled cystic spaces that lack both epithelial and endothelial linings [6]. Treatment modalities for aneurysmal bone cysts have traditionally been centered around surgical resection and intralesional curettage, with or without bone grafting. More recent approaches include adjuvant therapy such as cryotherapy, sclerotherapy, arterial embolization, and denosumab [3,6]. Denosumab is a monoclonal antibody that inhibits the receptor activator of nuclear factor kappa-B ligand (RANKL), a key regulator of osteoclastogenesis. Denosumab has demonstrated promising results in aneurysmal bone cysts, particularly in patients who are not suitable candidates for surgery or those with recurrent lesions. Here, we present the case of a 9-year-old boy diagnosed with an aneurysmal bone cyst who showed an excellent response to denosumab but experienced refractory rebound hypercalcemia and acute kidney injury after treatment discontinuation. The patient and his parents provided consent for the publication of this case report.

## 2. Case Presentation

**Figure 1:**
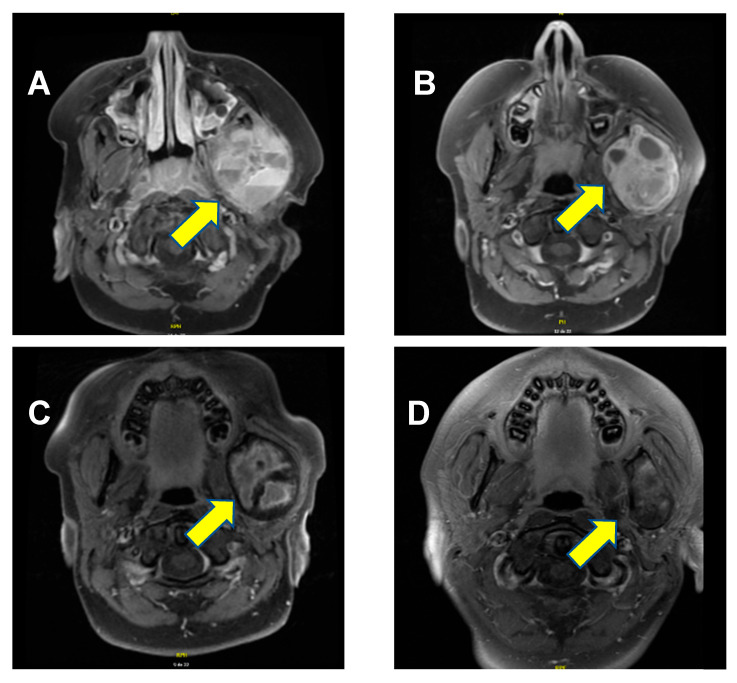
Axial T1-weighted MRI scan with gadolinium. (**A**) At diagnosis, a lesion of 5.2 cm × 4.6 cm × 4.1 cm was evidenced (arrow), containing multiple cystic components, with the bony cortex hard to establish and a relatively central enhancement. (**B**) Three months following the initiation of denosumab, the lesion measured 5.3 cm × 4.4 cm × 3.7 cm. The cystic components had diminished in size, enhancement appeared more important since the cystic parts were diminished, but the intensity of enhancement was similar. (**C**) About a month after administration of the last dose, the lesion measured 5.1 cm × 4.1 cm × 3.6 cm. Internal enhancement foci were still present, especially in the periphery. (**D**) Twenty-six months after treatment discontinuation, the lesion measured 4.3 cm × 2.6 cm × 3.5 cm. Post-contrast enhancement of signal anomalies was diminished on T2-weighting, showing that the radiological shrinkage of the lesion persisted.

A previously healthy 9-year-old boy presented with worsening edema of the left cheek that had developed over the course of several months. The patient had a visible and palpable lesion on the left branch of the jaw, which was mildly tender upon pressure. The mass was firm, and there was erythema. Of note, there were no other significant symptoms such as swallowing difficulties or difficulty breathing or speaking. The patient’s past medical history was entirely unremarkable. Notably, there was no evidence of intrauterine growth restriction or prematurity, and there were no clinical signs or family history of rickets. A cervical magnetic resonance imaging (MRI) confirmed the presence of a lesion measuring 5.2 cm × 4.6 cm × 4.1 cm located on the ascending branch of the left mandible, including the mandibular condyle. It presented several cystic components, measuring up to 4 cm× 2.6 cm. The masseter, temporal, and pterygoid musculature were lightly infiltrated by the lesion (Figure 1A). A temporomandibular biopsy was performed. The histopathology analysis showed bone and fibrous hemorrhagic fragments with evidence of fusiform mononuclear cells, as well as several multinuclear osteoclast-like cells, confirming the diagnosis of an aneurysmal bone cyst.

Treatment options were discussed with the patient and his family. Surgical resection was considered; however, the risk of morbidity was judged to be significant, prompting consideration of alternative treatment modalities. Considering the absence of any convincing alternative medical or surgical treatments and the disfiguring nature of the lesion, off-label treatment with subcutaneous denosumab was proposed, based on increasing anecdotal evidence of the drug’s efficacy in this setting [7,8,9]. Concerns about the risk of osteonecrosis of the jaw, as well as other potential side effects of this medication, were raised within the medical team in light of the scarce pediatric literature on the use of denosumab [8]. However, following multidisciplinary discussion and in agreement with the family, denosumab was deemed the best option.

Denosumab 2 mg/kg (120 mg) was administered subcutaneously once weekly for the first four weeks, then 2 mg/kg (120 mg) once a month for the subsequent sixteen months. This dose was selected based on previous experiences reported in the literature [10,11,12]. Supplementation therapy of calcium and vitamin D was given daily during the treatment to prevent hypocalcemia. Clinical response was rapid, with lesion pain resolution within a month. There was also a progressive decrease in the lesion’s dimensions after 17 months of treatment (Figure 1C).

The treatment was overall well-tolerated, aside from mild transitory myalgia. Following 17 months of treatment, there was a clinical and radiologic plateau in tumor reduction, which motivated the discontinuation of the treatment while maintaining careful follow-up, with monthly clinical and laboratory controls as well as MRI scans every three months. Approximately five months after the last denosumab administration, the patient presented to the emergency room with recurrent vomiting. Laboratory results disclosed severe hypercalcemia, with ionized calcium up to 2.07 (normal range: 1.04–1.37) mmol/L, and acute kidney injury, with creatinine up to 104 (normal range: 34–64, baseline values of this patient between 29 and 43) μmol/L, urea up to 9.2 (normal range: 2.6–7.2) mmol/L, and eGFR 53 mL/min/1.73 m^2^ (as per the simplified Schwartz equation). Further biochemical parameters are presented in Table 1.

An asymptomatic atrial flutter of about 7 s was also noted on cardiac monitoring, which was evaluated as secondary to hypercalcemia. It was an isolated, asymptomatic occurrence which spontaneously resolved with no further episodes. In collaboration with the endocrinology team, the patient was administered hyperhydration and four doses (day 1, 11, 14, and 19) of intravenous zoledronic acid (0.9 mg each, equivalent to 0.0125 mg/kg/dose), which resulted in only a mild and transient effect on calcium levels (Table 2).

In light of the persisting and significant hypercalcemia, with an understanding of the scanty available literature, interdisciplinary discussions between oncology and endocrinology were held. It was decided to administer a short course of prednisone (0.6 mg/kg once daily for three days and 0.5 mg/kg once daily for seven days), which resulted in a rapid and definitive resolution of hypercalcemia (Table 3) and recovery of kidney function. We hypothesize that the prednisone effect may have been mediated by steroid-induced hypercalciuria as well as decreased intestinal absorption of calcium. Moreover, previous literature suggests that corticosteroids are effective in managing hypercalcemia due to their ability to inhibit the CYP 27B1 enzyme, which converts 25-hydroxyvitamin D into its active form, 1,25-dihydroxyvitamin D [13,14]. Following interdisciplinary discussion between oncology and endocrinology specialists, we felt that the pathophysiological rationale and the favorable risk–benefit profile justified the decision to empirically proceed with this treatment. Alternatives approaches could have included the administration of one to two doses of denosumab to suppress osteoclast rebound, as well as the utilization of calcitonin; however, this drug was out of stock at our center. Fortunately, our patient did not require acute hemodialysis, which is reserved for more severe cases.

**Figure 2:**
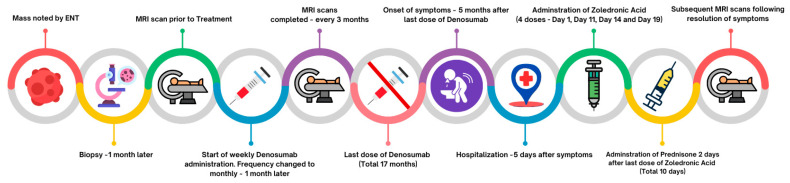
Timeline of patient’s clinical course from discovery of mass to regression of lesion and symptoms’ resolution.

Remarkably, the lesion continued to shrink even after denosumab discontinuation. At the most recent follow-up MRI, performed 26 months after treatment discontinuation, the lesion measured 4.3 cm × 2.6 cm × 3.5 cm, (Figure 1D). Most importantly, the significantly deformed malformation on the left aspect of the mandible is clinically no longer apparent; the aneurysmal bone cyst is meanwhile just barely palpable, and the patient no longer complains of any pain. A timeline of the patient’s clinical course, from initial discovery to lesion regression and symptom resolution, is provided in Figure 2.

## 3. Discussion

Aneurysmal bone cysts are a rare, benign RANK-ligand-mediated bone tumor that typically present in children and young adults and share many similarities with giant cell tumors of the bone (GCTB) [15]. In the absence of a standard treatment protocol for these patients [6], surgical resection is generally recommended whenever possible. The use of other treatments, such as high-speed burr, argon beam, intralesional doxycycline, phenol, percutaneous sclerotherapy, selective arterial embolization, intracystic injections of autologous bone marrow, and demineralized bone matrix, have been described [16,17,18]. Alternative treatment options are limited and include standalone radiotherapy, which is discouraged in this setting because of long-term side effects [17]. One clinical trial is currently evaluating sclerotherapy and bone marrow injection in aneurysmal bone cysts (NCT05696834). Furthermore, another ongoing trial is investigating the role of Discogel^®^, a sclerosis agent, in the treatment of aneurysmal bone cysts (NCT05880628).

Denosumab is a human monoclonal antibody that links to the cytokine receptor activator of nuclear factor-kappa B ligand (RANKL), thereby decreasing osteoclast activity [7]. This drug is generally used to treat osteoporosis, particularly in postmenopausal women at high risk of fractures [19].

Given this mechanism of action, there have been several attempts at denosumab treatment for aneurysmal bone cysts (ABCs) in children. Data on its efficacy; however, remains limited and there are concerns with respect to treatment-related toxicity [7,20]. Indeed, some significant drug-related side effects have been reported, most prominently hypercalcemia at discontinuation [9,17,21,22,23,24,25,26]. The underlying mechanism is thought to be caused by the rebound reactivation and formation of osteoclasts, which results in a rapid loss of newly formed bone when denosumab is stopped [7,24,26]. In the herein reported case, hypercalcemia ensued five months after the last denosumab dose. The reason for this latency is unclear, although it does not appear exceptional; indeed, hypercalcemia has been encountered up to nine months after denosumab discontinuation [26,27,28,29,30].

In most reported cases, hypercalcemia resolved with bisphosphonates [21,22,23,25,26,29,30]. Although prednisolone was effective in a patient, its prolonged use resulted in Cushing syndrome [21]. In contrast to this previous experience, the patient herein reported experienced only a brief benefit from bisphosphonate treatment, while a short course of prednisone successfully and permanently resolved the hypercalcemia without any significant toxicity. Although we cannot rule out the possibility that the improvement was due to a combination of treatments and cumulative doses, the rapid response to steroids was striking, with complete resolution of the previously refractory hypercalcemia.

From a therapeutic perspective, our patient exhibited an excellent response to denosumab, with persisting ongoing reduction in the mass 26 months after treatment discontinuation (Figure 1D). The fact that this initial deforming lesion is no longer clinically noticeable has had a significant impact on this child’s self-esteem and quality of life. Of course, close clinical monitoring and follow-up remain essential, as there are reports of tumor recurrence after denosumab discontinuation [31,32].

In retrospect, at the end of denosumab treatment, the patient might have benefited from preventive administration of bisphosphonates and/or of a progressive tapering of denosumab instead of an abrupt discontinuation, in an effort to try and prevent (or limit) the potential metabolic side effects [15]. These may indeed be useful learning points derived from our experience and might benefit future patients.

It is important to stress that the herein reported patient will need long-term follow-up due to the possible denosumab-related late side effects, specifically in regard to bone health and dental development [33], on one side, and to promptly identify any clinical or radiological signs of disease progression, on the other. At the time of publication, the patient continues to undergo MRI and laboratory assessments, currently scheduled at six month intervals, as well as regular endocrinology clinics.

Two interesting questions arise. First, if the patient were to develop an increase in lesions size, should a second denosumab course, or even a maintenance therapy, be initiated? Given the striking response to the first course but also the significant side effects, the answer is tricky and will need a careful benefit–risk assessment. Second, whether a maintenance therapy with a different drug might be useful (to avoid future increase in lesion size) is interesting as well, but remains without clear-cut answer. In this case, for the time being, we elected for a conservative approach of close monitoring.

This case report obviously suffers from some inherent limitations. First of all, it is just a single observation without any clear-cut causal relationship regarding both efficacy and the hypercalcemia side effect, which is assumed but not proven. However, the temporal relationship, in conjunction with pathophysiological rationale and the otherwise expected natural history, supports a potentially causal role with respect to efficacy. With regard to hypercalcemia, we calculated a Naranjo score of 3, which allows to classify a causal association as possible. Second, alkaline phosphatase and urinary calcium excretion, which might have been useful in the diagnostic algorithm of hypercalcemia, were regrettably not assessed at hypercalcemia presentation. Finally, further limitations of this case report include its retrospective nature, and the absence of genetic analyses to rule out potentially inherited bone diseases, which could have guided the decision-making process.

## 4. Conclusions

Aneurysmal bone cysts are rare and benign lesions that have limited therapeutic options. Currently, there is no “gold standard” recommended treatment. Denosumab has emerged as a promising off-label drug in this situation. However, its use carries the risk of significant side effects, including rebound hypercalcemia and acute kidney injury. Considering the benign character of this tumor, the decision to use denosumab for aneurysmal bone cysts should be evaluated on a case-by-case basis by a multidisciplinary team, with careful balancing of benefits and risks. This case report highlights that, if denosumab is elected for treatment, clinicians should carefully monitor calcium levels and kidney function for several months after discontinuing the drug. There are currently no recommendations on frequency and timing of monitoring. We would propose weekly checks during the first month and monthly checks for the subsequent six months. Moreover, preventive administration of bisphosphonates and a progressive tapering of denosumab are strategies that should be explored and considered.

## Figures and Tables

**Table 1:** Biochemical parameters before denosumab treatment, during denosumab treatment, at presentation with hypercalcemia, and after hospitalization.

Assessed Parameter (Normal Range)	Pre-Denosumab	During Treatment	At Presentation with Hypercalcemia	After Hospitalization
Calcium (2.20–2.65 mmol/L)	2.52 mmol/L	Between 2.10 mmol/L and 2.57 mmol/L	3.17 mmoL/L	Between 2.35 mmol/L and 2.95 mmol/L
Ionized calcium corrected for 7.4 pH (1.14–1.32 mmol/L)	-	1.35 mmol/L	2.01 mmol/L and 2.07 mmol/L	Between 1.28 mmol/L and 1.49 mmol/L
Albumin (37–47 g/L)	39 g/L	Between 38 g/L and 41 g/L	32 g/L (day after presentation)	Between 38 g/L and 40 g/L
Phosphate (1.30–1.80 mmol/L)	1.57 mmol/L	Between 0.66 mmol/L and 1.50 mmol/L	1.09, 0.96, and 0.76	Between 0.87 and 1.37
Magnesium (0.70–1.05 mmol/L)	0.97 mmol/L	Between 0.88 mmol/L and 1.07 mmol/L	0.67 mmol/L and 0.63 mmol/L	Between 0.81 mmol/L and 0.94 mmol/L
25-hydroxy vitamin D (50–125 nmol/L)	56.4 nmol/L	-	60.6 nmol/L	Between 52.9 nmol/L and 75.4 nmol/L
PTH I-84 (15–68 ng/L)	51 ng/L	-	<5 ng/L	-
Creatinine (25–75 µmol/L)	43 µmol/L	Between 29 µmol/L and 43 µmol/L	84 µmol/L, 88 µmol/L, and 93 µmol/L	Between 35 µmol/L and 68 µmol/L
Urinary calcium/urinary creatinine (0.04–0.70 mmol/mmol)	-	-	-	0.64

**Table 2:** Days of zoledronic acid administration and associated total and ionized calcium levels.

Zoledronic Acid Dose Administration	Calcium (2.20–2.65 mmol/L) Before Zoledronic Acid Dose Administration	Calcium, Approximately 2 Days After Dose Administration	Ionized Calcium Corrected for pH (1.14–1.32 mmol/L) Before Zoledronic Acid Dose Administration	Ionized Calcium Corrected for pH (1.14–1.32 mmol/L) Approximately 2 Days After Zoledronic Acid Dose Administration
Day 1	3.17 mmol/L	-	2.07 mmol/L	1.42 mmol/L
Day 11	3.08 mmol/L	3.17 mmol/L	1.60 mmol/L	1.84 mmol/L
Day 14	3.17 mmol/L	2.77 mmol/L	1.84 mmol/L	1.64 mmol/L
Day 19	3.19 mmol/L	3.04 mmol/L	1.62 mmol/L	-

**Table 3:** Time course of total and ionized calcium before, during, and after prednisone administration.

Date	Calcium (2.20–2.65 mmol/L)	Ionized Calcium Corrected for pH 7.4 (1.14–1.32 mmol/L)
Pre-prednisone	3.04 mmol/L	1.62 mmol/L
During prednisone	Between 2.28 mmol/L and 2.95 mmol/L	1.32 mmol/L
Approximately one month post-prednisone	2.62 mmol/L	1.43 mmol/L
Approximately two months post-prednisone	2.52 mmol/L	1.33 mmol/L

## Data Availability

The data presented in this study are available on reasonable request from the corresponding author. The data are not publicly available due to anonymity considerations.

## Abbreviations

The following abbreviations are used in this manuscript:

ABC	Aneurysmal bone cysts
GCTB	Giant cell tumors of the bone
MRI	Magnetic resonance imaging
RANKL	Receptor activator of nuclear factor kappa-B ligand
